# Signal integrator function of CXXC5 in Cancer

**DOI:** 10.1186/s12964-024-02005-x

**Published:** 2025-01-14

**Authors:** Zihao An, Jiepu Wang, Chengzuo Li, Chao Tang

**Affiliations:** National Clinical Research Center for Child Health of Children’s Hospital, Zhejiang University School of Medicine, Hangzhou, 310052 China

**Keywords:** CXXC5, Cell signaling, Cancer, Targeting therapy

## Abstract

CXXC type zinc finger protein 5 (CXXC5) is a member of the ZF-CXXC family and plays a pivotal role in signal integration and information transfer within cell signaling network. CXXC5 acts as a regulator in various physiological processes, and abnormalities in its protein structure or function have been linked to multiple pathological processes. In this article, we correspondingly describe the composition of the ZF-CXXC family, emphatically introducing the features of the CXXC5 gene and protein, review the role of CXXC5 in cellular signaling networks, the physiological and pathological processes associated with CXXC5 dysregulation, and particularly focus on the correlation between CXXC5 and cancers. Finally, we summarize the current therapies targeting CXXC5 and their potential applications, and discuss the intriguing findings from current studies, and the opportunities and challenges in future.

**Graphical Abstract:**

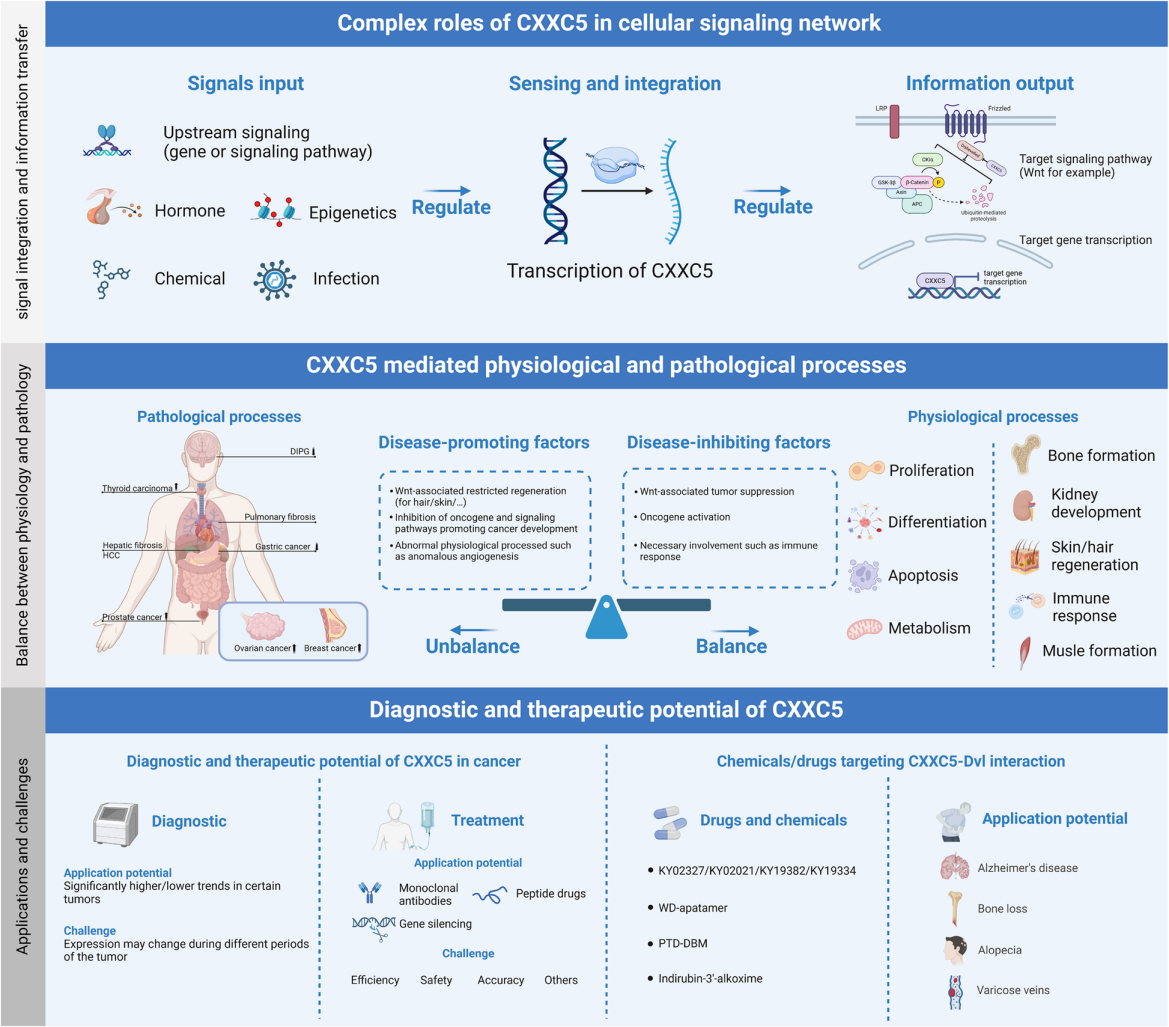

## Introduction

CXXC type zinc finger protein 5 (CXXC5), also named RINF or WID, is a member of the ZF-CXXC protein family. Members of this protein family share a conserved ZF-CXXC domain capable of binding to non-methylated cytosine and guanine dinucleotides (CpGs), thereby exerting transcriptional regulation or epigenetic regulation. Unlike other family members, CXXC5 lacks intrinsic catalytic activity and requires interaction with other epigenetically regulators to achieve its epigenetic regulatory function [1, 2]. CXXC5 is widely expressed in human tissues, and its expression or function is regulated by multiple intra- and extracellular factors, including hormones, oxygen concentration, and various signaling pathways. CXXC5 is associated with several signaling pathways, including Wnt/β-catenin, TGF-β/BMP, ATM/p53, and PI3K-Akt, and it also participates in the transcriptional regulation and epigenetic modification of genes such as *MBP*, *p21CIP1*, *ACVR1*, *Flk-1*, and *MYC1* [3–9]. This complex up- and downstream signaling network allows CXXC5 to exert a wide range of regulatory effects on cell proliferation, differentiation, apoptosis, and metabolism, among others. Furthermore, CXXC5 plays a role in physiological processes such as growth and development, angiogenesis, and hair regeneration. Abnormalities in the expression or function of CXXC5 are linked to pathological conditions such as cancer development, restricted hair regeneration, and abnormal fibrosis.

Here, we provide a brief overview of the composition and characteristics of the ZF-CXXC family, as well as the roles and functions of CXXC5 within this family, with a focus on the integration and transduction function of CXXC5 in complex intra- and extracellular signaling networks, including the factors that regulate the expression and function of CXXC5 as well as the role of CXXC5 in the regulation of cellular signaling pathways, transcription, and epigenetics. Furthermore, we describe the role of CXXC5 in various physiological and pathological processes, in particular, its role in the development of cancer. Finally, we briefly summarize the emerging therapeutic modalities through the modulation of CXXC5 function and highlight the key unresolved issues related to CXXC5 and future research directions.

## ZF-CXXC protein family: modifiers of unmethylated CpG islands

The ZF-CXXC protein family was discovered and defined during the process of attempting to find proteins capable of binding to unmethylated CpG islands (CGIs) [1, 2]. This family exhibits a conserved ZF-CXXC domain capable of interacting with unmethylated CGIs and plays a significant role in epigenetic regulation, either through its intrinsic catalytic activity or by recruiting other epigenetically regulators [1]. Based on the differences in the ability to combine motifs, the ZF-CXXC domains (residues 250–306) in humans can be classified into four subgroups: CpGpG binding subgroup, CpG binding subgroup, CpH (H refers to any non-G nucleotide) binding subgroup, weak or no CpG binding ability subgroup [2, 10, 11]. The ZF-CXXC domain comprises two distinctive and conserved CXXCXXC motifs (i.e., CxxCxxCx4-5CxxCxxC and CxxRxC, where x represents residues other than cysteine) that coordinate two Zn^2^⁺ ions, based on their sequence similarity, the ZF-CXXC protein family can be divided into three subgroups in mice: the type-1 subgroup contain KDM2A, KDM2B, FBXL19, CFP1, DNMT1, MLL1, MLL2, and MBD11; the type-2 subgroup contain MBD12 and MBD13; the type-3 subgroup contains TET1, TET3, IDAX and CXXC5 (summarized in Table 1) [1].

**Table 1:**
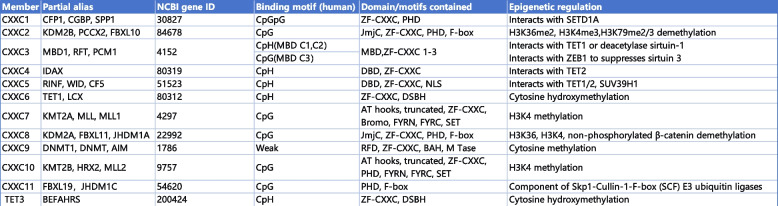
Members of ZF-CXXC protein family [1, 2, 10, 11]

## CXXC5: from gene to protein

**Fig. 1:**
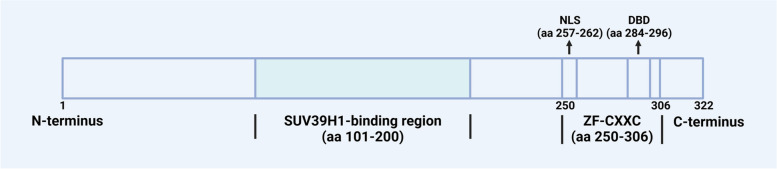
Shematic representation of CXXC5 protein structure [19, 20, 22]

The *CXXC5* gene is evolutionarily conserved, with a length of 35.5 kbp, and localized on human chromosome 5q31.2, where it is close to regions frequently deleted in patients with acute myeloid leukemia (AML) or myelodysplastic syndrome (MDS) and found to be an abnormal fusion partner with *MN1* [12–15]. The *CXXC5* comprises 11 exons and 10 introns, with the A segment of exon 3 representing the CXXC5 core promoter element, which is situated within the CGI [16]. There are 14 transcript variants of CXXC5 found in humans encoding the identical CXXC5 protein and are expressed differently in different tissues; among these, transcript variant 2 exhibits the highest expression level among the variants and represents the principal transcript in cell models [16]. Differences in transcripts may be associated with cardiac physiological and pathological hypertrophy [17]. The CXXC5 protein contains 322 amino acids in length with a molecular weight of 32.98 kDa and exhibits a high degree of disorder at its N-terminus. The ZF-CXXC domain of CXXC5 belongs to the type-3 ZF-CXXC domain, which is closely related to the function of CXXC5 [1, 2, 10]. In addition to possessing CpH-binding activity, which is the basis for the epigenetic and transcriptional regulation of CXXC5, this domain also participates in the regulation of Wnt/β-catenin, TGF-β, and ATM-p53 signaling pathways by CXXC5. The ZF-CXXC domain is essential for the interactions of CXXC5 with Smad2/3, as well as the DNA damage-induced ATM phosphorylation [18, 19]. Furthermore, CXXC5, which lacks the ZF-CXXC domain, is unable to inhibit Wnt signaling despite retaining the capacity to bind Dvl [20]. In addition, the "KTXXXI" motif (X refers to any amino acid) is the smallest disheveled (Dvl) binding domain (DBD) in CXXC4, and the CXXC5-Dvl interaction can be hindered by deletion of the putative DBD (residues 284-296) in CXXC5 [20, 21]. Moreover, the C-terminus of CXXC5 contains a nuclear localization sequence (NLS, residues 257-262), which is necessary for the nuclear localization ability of CXXC5 and for ATM phosphorylation after DNA damage [19], and the middle region of CXXC5 (residues 101–200) is essential for the interaction between CXXC5 and SUV39H1 (shown in Fig. 1) [22].

## CXXC5 in signal transduction network: signal integrator and information transfer

**Fig. 2:**
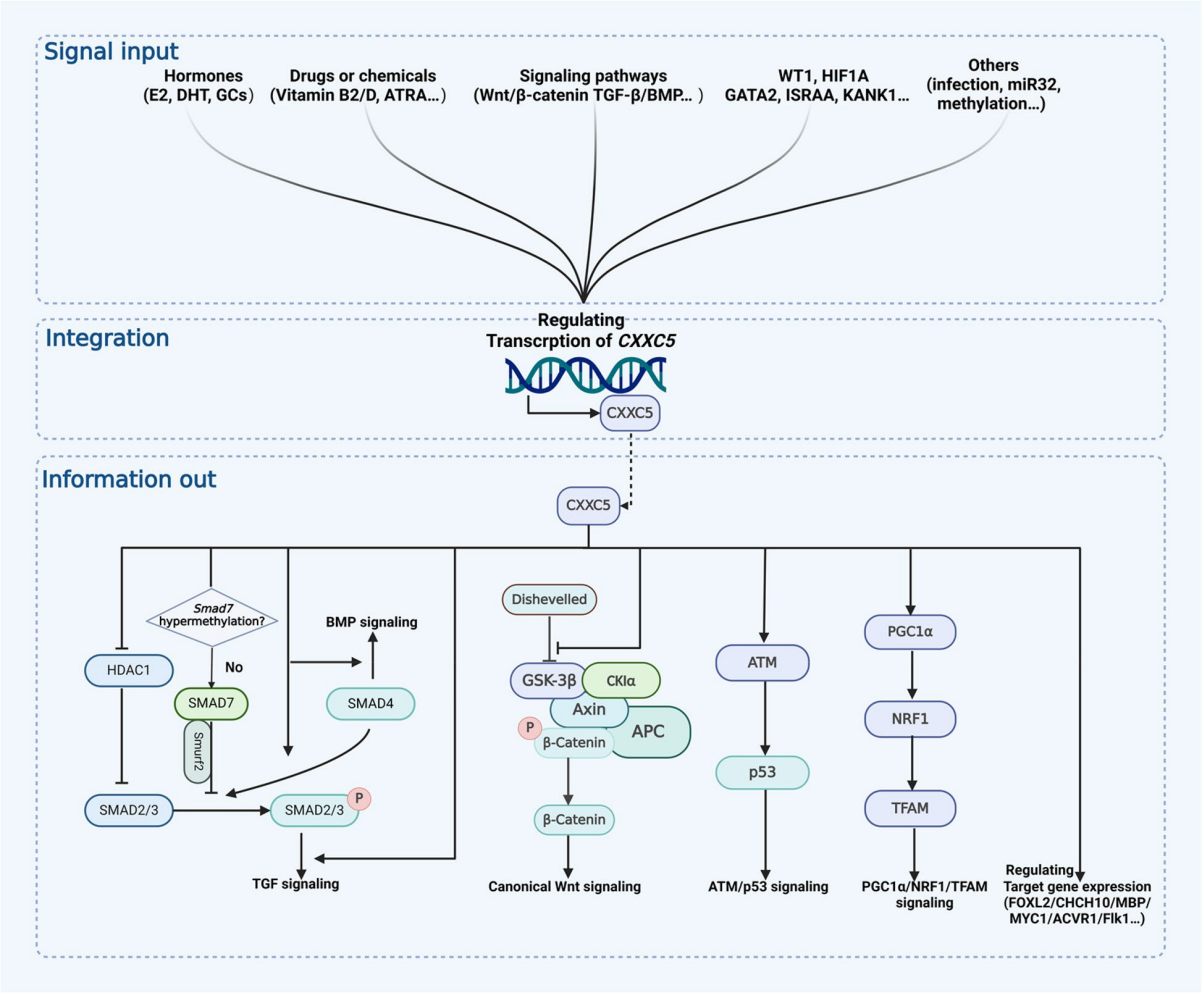
Signal integration and information transfer functions of CXXC5

CXXC5 is pivotal in signal integration and information transfer in the cellular signaling network. The expression and function of CXXC5 are regulated by complex factors from both intrinsic and extrinsic to the cell, which in turn affect the transduction of multiple intracellular signal pathways and the expression of multiple genes downstream through the transcriptional and epigenetically regulatory functions of CXXC5 (shown in Fig. 2).

### Upstream signals regulating CXXC5

Multiple intra- and extracellular signals are involved in the regulation of CXXC5 expression. As a crucial effector of these signals, CXXC5 is intimately associated with the physiological regulation and pathological states they mediate.

#### Signaling pathways

Wnt/β-catenin and TGF-β/BMP signaling have been identified as important positive regulators of CXXC5. In human colon cancer cells, CXXC5 expression is upregulated by β-catenin, and the β-catenin/T-cell factor 4 (TCF4) complex has been discovered to coordinate with a chromatin loop integrated 5' and 3' Wnt responsive DNA enhancers (WREs) [7]. The activation of CXXC5 by Wnt/β-catenin signaling was also observed in neural stem cells (NSCs), which is required for oligodendrocyte differentiation and myelin production [8]. In NSCs, BMP4 can directly induce CXXC5 expression, which is essential for NSC differentiation induced by BMP4 signaling [3]. Induction of CXXC5 by BMP4 was also observed in human umbilical vein endothelial cells (HUVECs), where the protein level of CXXC5 is increased in a time-dependent manner with increasing level of phosphorylated Smad1/5/8. In this process, the expression of CXXC5 is essential for BMP4-mediated angiogenesis [4]. Furthermore, Induction of CXXC5 expression by TGF-β1/BMP signaling has also been observed in Hep3B, HepG2, and diffuse intrinsic pontine glioma (DIPG) cell lines [5, 6].

#### Hormones

CXXC5 seems to be a novel and crucial effector of hormones. In MCF7 cells, which belong to the estrogen receptor (ER) positive human breast cancer (BC) cell line with a positive response to estrogen induction, ERα-E2 (17β-estradiol) directly upregulates CXXC5 expression through the interaction with estrogen response element (ERE), and CXXC5 subsequently regulates the expression of downstream genes in an estrogen-dependent or independent manner [23, 24]. In human chondrocyte cell line C28/I2, E2 has been observed to significantly increase CXXC5 expression and play an important role in estrogen-mediated growth plate senescence, and this effect was not observed significantly in *Cxxc5*^*−/−*^ mice even when given estrogen stimulation [25]. In addition, dihydrotestosterone (DHT) has been discovered to upregulate CXXC5 expression via the DHT-PGD2-Smad1/5/9 axis in human immortal keratinocyte line (HaCaT), thereby inhibiting Wnt signaling via CXXC5-Dvl interaction and mediating androgenetic alopecia [26]. Interestingly, knockdown of androgen receptor (AR) in human prostate cancer (PCa) C4-2 cells has been observed to promote CXXC5 expression [27]. Further investigation is required to elucidate the relationship between CXXC5 and androgens and androgen receptors. While a microarray experiment has indicated that glucocorticoids (GCs) may act as a negative regulator of CXXC5 in the CNS, but the underlying mechanisms remain unclear [28].

#### Drugs or chemicals

The expression of CXXC5 can be influenced by certain drugs or chemicals, and their own effects are also affected by CXXC5. All-trans retinoic acid (ATRA) can directly induce the expression of CXXC5, which has been shown to play an important role in ATRA-induced terminal differentiation of acute promyelocytic leukemia (APL) cells [29]. This is the origin of CXXC5 being called RINF (retinoid-inducible nuclear factor). In addition, up-regulation of CXXC5 expression was also observed in DIPG cells treated with histone deacetylase (HDAC) inhibitors (dacinostat, quisinostat, and panobinostat) [6]. Tetrachlorodibenzo-p-dioxin (TCDD), a dioxin-like environmental pollutant, exhibits the capacity to suppress CXXC5 expression through AHR response elements (AHREs) [30]. Furthermore, vitamin D and vitamin B2 have been observed to affect CXXC5 expression, the exact mechanism remains elucidated [31].

#### Other factors

Some immune-related factors, including T-helper-inducing POZ/Krueppel-like factor (ThPOK) and immune-system-released activating agent (ISRAA), *P. gingivalis* infection and IL-6 also connects with the expression of CXXC5 [22, 32–34]. Additionally, specific regulatory factors including NUDT21, SDF-1/CXCR4, ELF1/MAZ, EZH2, microRNA-32, and KANK1, can also regulate the expression or function of CXXC5 directly or indirectly [16, 35–40].

Furthermore, disparities in CpG methylation within the CXXC5 promoter were identified in prostate, bladder, and thyroid cancers compared to normal tissues, indicating that methylation may play a role in regulating CXXC5 expression [41–43]. It is noteworthy that several studies have reported that the regulation of CXXC5 expression is context-dependent. In MCF7 cells, the overexpression of MAZ was observed to promote CXXC5 expression; however, silencing of MAZ did not result in the inhibition of CXXC5 expression, suggesting that stable conditions may prevent MAZ from regulating CXXC5 [16]. Moreover, it was observed that TGF-β exposure enhanced CXXC5 expression in the normal hepatocyte cell line HL-7702 and several HCC cell lines, including Hep3B, HepG2, and Huh7. However, this effect was not found in MHCC97L or MHCC97H cells [5]. In addition, the temporal regulation of CXXC5 by E2 has been observed. In MCF7 cells, CXXC5 was observed to be upregulated at 3 h and 24 h but not at 6 h following treatment with E2, and this phenomenon waits to be further discovered [24] (summarized in Table 2).

**Table 2:**
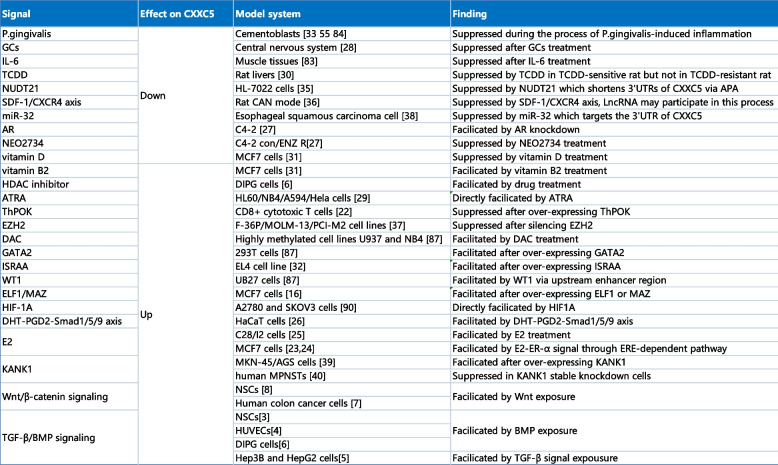
Signals regulating the expression of CXXC5 [3, 4, 6, 6–8, 16, 23–27, 27–31, 31–33, 35, 35–37, 37, 38, 38–40, 44–47, 47, 47, 48]

*Abbreviations*: *TCDD* Tetrachlorodibenzo-p-dioxin, *AR* Androgen receptor, *E2* 17β-estradiol, *ERα* Estrogen receptor α, *HaCaT* Human immortal keratinocyte line, *CAD* Chronic allograft nephropathy, *ENZ* Enzalutamide, *DIPG* Diffuse intrinsic pontine glioma, *NSCs* Neural stem cells

### Mechanisms of CXXC5

The ZF-CXXC family plays a vital role in epigenetic modification. Unlike other family members, CXXC5 lacks intrinsic catalytic activity and relies on interactions with other epigenetically regulators, including SUV39H1, Tet1, Tet2, and so forth, to exert epigenetic regulation [22, 27, 49, 50]. Furthermore, CXXC5 functions as a coordinator of multiple cellular signaling pathways, participating in the regulation of signaling pathways such as Wnt/β-catenin and TGF-β/BMP through interactions with Dvl, HDAC1, Smad, and others [5, 18, 51–53]. Additionally, CXXC5 is also involved in the regulation of gene expression as a transcription factor. Notably, it has recently been suggested that CXXC5, which also lacks transcriptional activation/repression activity, participates in the formation of restrictive or permissive chromosomal states for transduction as a scaffold protein [23, 54]. To better understand the mechanism of action of CXXC5, Table 3 lists its gene-binding regions and interacting partners of CXXC5, along with their effects on transcription, epigenetic, and cellular signaling pathways.

**Table 3:**
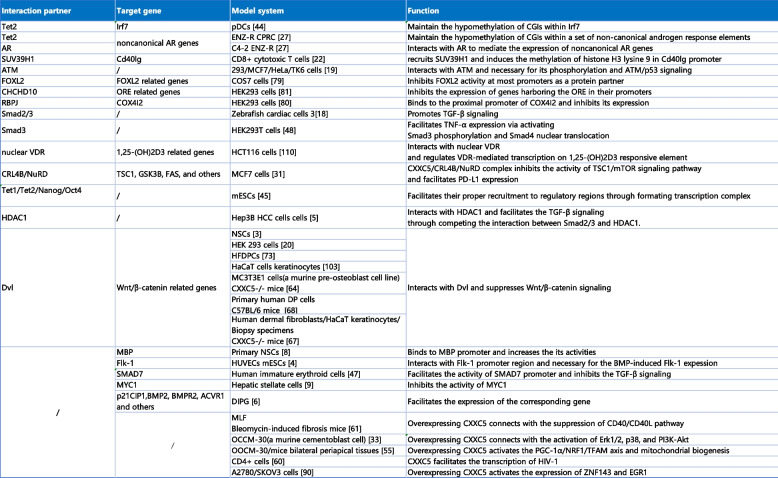
CXXC5 in signal transduction network [3–6, 8, 9, 18–20, 22, 27, 27, 31, 33, 44, 48–50, 52, 53, 55–59, 59–64]

*Abbreviations*: *pDCs* plasmacytoid dendritic cells, *MLF* Mouse lung fibroblast, *mESCs* mouse embryonic stem cells, *ENZ-R* Enzalutamide resistance, *VDR* Vitamin D receptor, *DP* Dermal papilla, *HFDPCs* Human hair follicle dermal papilla cells, *COX4I2* Cytochrome c oxidase isoform 2, *ORE* Oxygen responsive element, *1,25(OH)2D3* 1,25-dihydroxyvitamin D3

### The regulatory function of CXXC5

#### CXXC5 in Wnt/β-catenin signaling pathway

CXXC5 is an important negative feedback factor of the Wnt/β-catenin signaling pathway. The Wnt/β-catenin signaling pathway, also known as the canonical Wnt pathway, plays an important role in cell proliferation and tissue homeostasis [65]. Dvl is located in the cytoplasm and plays an essential role in the transduction of Wnt signaling to the intracellular compartment [66]. As previously stated, Wnt signaling promotes CXXC5 expression in human colon cancer cells and NSCs, and the up-regulated CXXC5 can prevent the nuclear translocation of β-catenin and the transcription of related target genes by interacting with Dvl, thereby forming a negative feedback loop for Wnt signaling [7, 8, 51]. Several studies have discovered this phenomenon involving NSCs, HEK 293 cells, and other cells and tissues (summarized in Table 3) [3, 20]. Furthermore, the activation of Wnt signaling by targeting the CXXC5-Dvl interaction has been identified as a significant factor in several different cells and tissues. The activation of Wnt signaling by targeting CXXC5-Dvl interaction has potential clinical applications in skin healing, hair regeneration, angiogenesis, and others (summarized in Table 4).

#### CXXC5 in TGF-β/BMP signaling pathway

CXXC5 acts as a feedback regulator in the TGF-β/BMP signaling pathway. The TGF-β/BMP signaling pathway plays an essential role in regulating several crucial biological processes, including bone development and angiogenesis, and the Smad protein is a vital element of the TGF-β/BMP signaling pathway, which is intimately associated with the transcription of downstream target genes [67, 68]. The TGF-β/BMP signaling pathway has been discovered to enhance CXXC5 expression in HUVECs, NSCs, Hep3B, HepG2, DIPG, and many other cells and tissues [3–6]. The effect of CXXC5 on the TGF-β/BMP signaling pathway is achieved primarily through its influence on the expression and function of the Smad protein. In Hep3B cells, competitive binding of CXXC5 to HDAC1 has been demonstrated to attenuate its inhibition of the TGF-β/BMP signaling pathway through releasing Smad2/3 [5]. In HEK293T cells, CXXC5 interacts with Smad3, affecting the expression of downstream genes by promoting the phosphorylation of Smad3 and the nuclear translocation of Smad4 [53].

Similarly, in zebrafish cardiac cells, CXXC5 was found to interact with Smad2/3 and promote the TGF-β/BMP signaling pathway [18]. The studies above collectively indicate that CXXC5 can function as a positive feedback regulator, promoting the TGF-β/BMP signaling pathway.

Notably, the influence of CXXC5 on the TGF-β/BMP pathway appears to extend beyond this conclusion. It has recently been demonstrated that the expression of CXXC5 can adjust the response of immature human erythroid cells to the TGF-β/BMP signaling pathway through the direct up-regulation of Smad7 expression, which is an inhibitory protein of the TGF-β/BMP signaling pathway [52]. Indeed, this finding does not contradict the previously concluded “CXXC5 as a positive feedback regulator”. The *Smad7* promoter is silenced in non-hematopoietic tissues due to hypermethylation, which may explain why CXXC5 does not exhibit Smad-7-dependent inhibition of the TGF-β signaling in most non-hematopoietic tissues [69].

#### CXXC5 in other signaling pathways and genes expression regulation

CXXC5 has also been implicated in the regulation of several other signaling pathways and gene expression. CXXC5 interacts with ATM and is essential for the phosphorylation of ATM induced by DNA damage and the ATM/p53 signaling [19]. Furthermore, overexpression of CXXC5 in OCCM-30 was observed to result in the activation of the Erk1/2, p38, and PI3K-Akt signaling pathways. Conversely, silencing of CXXC5 led to the inhibition of these pathways [33]. Similarly, overexpression of CXXC5 in OOCM-30 promoted the PGC-1α/NRF1/TFAM axis and mitochondrial biogenesis [44]. Moreover, the interaction of CXXC5 with NuRD/CRL4B in BC cells can inhibit TSC1/mTOR signaling and promote PD-L1 expression [31]. In addition, CXXC5 has been demonstrated to regulate the expression of several other genes, including Irf7, MBP, Flk-1, MYC1, p21CIP1, BMP2, BMPR2, ACVR1, and others, which involves the regulation of complex cellular signal transduction networks by CXXC5 (summarized in Table 3) [4, 6, 8, 9, 49].

## CXXC5 in physiological and pathological processes

CXXC5 is widely expressed in various human tissues and is pivotal in the cell signaling network, facilitating signal integration and information transfer [29, 51]. This broad expression and its pivotal role make CXXC5 closely linked to multiple cellular manners, including metabolism, proliferation, differentiation, and apoptosis [53, 70, 71]. Correspondingly, the appropriate regulation of CXXC5 and these cellular manners are intimately associated with various physiological processes, including bone formation and immune system regulation; conversely, abnormalities in CXXC5 expression or function have been linked to a wide range of pathological processes and diseases, including bone loss, infection, and especially cancers [25, 49, 55, 72, 73].

### CXXC5 in cell proliferation, differentiation, and tissue generation

CXXC5 is a crucial regulator of cell proliferation, differentiation, and tissue generation. Given the intricate role of CXXC5 in the cell signaling network, its regulatory pathway in these processes is similarly complex. However, it can be divided into two main categories: the Wnt-associated pathways and the non-Wnt-associated pathways. CXXC5 plays a pivotal role in the growth and development of tissues, and it is also involved in pathological processes such as *P. gingivalis* infection and fibrosis [33, 44, 56].

#### Wnt associated pathways

The regulation of Wnt signaling by CXXC5 profoundly impacts proliferation and differentiation behaviors in many tissues and cells, including oligodendrocytes differentiation, as well as the development of telencephalon and kidney. During neurological development, CXXC5 has been demonstrated to promote the differentiation of oligodendrocytes and the production of myelin; *Cxxc5*^*−/−*^ mice exhibit severely reduced expression of myelin basic protein and abnormal myelin structure [8]. Furthermore, CXXC5 expression is induced by BMP4 and inhibits Wnt3a signaling by interacting with Dvl2 during mouse telencephalon development; this regulation contributes to delineating BMP4 and Wnt3a signaling regions and maintaining normal developmental behaviors during mouse telencephalon development [3]. Similarly, CXXC5 and its upstream activators are co-expressed in podocytes of maturing nephrons during zebrafish kidney development. The ablation of CXXC5 can result in abnormal kidney development and the formation of large cysts in the glomerular-tubular regions of zebrafish embryos [20]. Additionally, it was demonstrated that activated Wnt signaling also results in the formation of cystic kidneys in zebrafish and mice, consistent with the discoveries that CXXC5 can inhibit Wnt signaling [74, 75].

Furthermore, negative feedback regulation of the Wnt signaling pathway by CXXC5 plays an essential role in bone formation, angiogenesis, skin repair, hair regeneration, and adipocyte differentiation. During osteoblast and bone differentiation, *Cxxc5*^*−/−*^ mice exhibited high bone mass phenotypes as well as increased osteocyte dendrite formation and bone formation rate, and osteoblast differentiation and ex vivo calvaria growth can be promoted by blocking the CXXC5-Dvl interaction as well as reducing bone loss in ovariectomized (OVX) mouse models [57, 72]. In addition, CXXC5 expression is progressively elevated during the senescence process in rodent growth plates, and elevated CXXC5 expression inhibits Wnt signaling by interacting with Dvl, suppressing Wnt-regulated molecules related to chondrocyte maturation transcription. *Cxxc5*^*−/−*^ mice exhibit delayed growth plate senescence and tibial elongation [25].

CXXC5 participates in the regulation of diabetic wound healing and angiogenesis, and blocking the Dvl-CXXC5 interaction to activate the Wnt signaling pathway can enhance angiogenesis and skin repair in diabetic mice [76, 77]. Moreover, a reduction in CXXC5 protein expression is evident in epidermal keratinocytes and skin fibroblasts from acute wounds in humans, and the blockade of the CXXC5-Dvl interaction was observed to promote hair healing, with *Cxxc5*^*−/−*^ mice exhibiting faster skin healing [58, 59, 78, 79]. Similarly, CXXC5 is expressed in keratin-forming cells of hair follicles, hair follicle dermal papilla cells, and other hair production-related cells. CXXC5 inhibits the Wnt signaling pathway and suppresses the process of follicle regeneration by interacting with Dvl, and accelerated hair regeneration can be observed in *Cxxc5*^*−/−*^ mice [26, 80–82]. The mRNA level of CXXC5 was found to be higher in differentiated adipocytes in comparison to preadipocytes from the human omentum and subcutaneous adipose tissue, and up-regulated CXXC5 has been observed to promote adipocyte differentiation by inhibiting Wnt signaling [83].

#### Non-Wnt-associated pathways

In addition to the CXXC5-Dvl interaction, CXXC5 exerts regulatory effects on cardiogenesis, angiogenesis, skeletal myogenesis, myelopoiesis, fibrosis, and proliferation or differentiation of embryonic, cementoblasts, and myeloid cells through other pathways.

CXXC5 is consistently expressed during cardiogenesis and regulates cardiac development and circulation through the TGF-β signaling pathway; knockdown of CXXC5 during zebrafish cardiac development has been observed to result in looping defects, cardiac dysplasia, pericardial edema, and other cardiac developmental abnormalities [18]. Furthermore, in mouse embryonic stem cells, CXXC5 forms a complex with Tet1, Tet2, Nanog, and Oct4, which positively regulates the transcription of pluripotency genes and Tet enzymes and ensures the normal differentiation of embryonic stem cells [50]. CXXC5 binds directly to the *Flk-1* promoter region and promotes Flk-1 transcriptional activity; correspondingly, CXXC5 is required for BMP signaling-induced Flk-1 expression, motility, and tube formation in endothelial cells; conversely, aberrant expression of CXXC5 results in impaired venous angiogenesis during zebrafish development and the down-regulation of CXXC5 was also observed in vein wall tissue from patients with varicose veins (VV) [4, 84]. In the context of skeletal muscle cell differentiation, it has been found that CXXC5 can significantly increase the activity of the promoters of genes related to skeletal muscle differentiation in C2C12 myoblasts and promote skeletal muscle differentiation [85]. CXXC5 regulates the differentiation process of cementoblasts through multiple pathways, including p38, PI3K-Akt, Erk-1/2, and PGC-1α. *P. gingivalis* infection inhibits CXXC5 expression, thereby inhibiting cementoblasts differentiation [33, 44].

In the hematopoietic system, CXXC5 plays a vital role in normal bone marrow hematopoiesis, and during myeloid differentiation of normal CD34 + progenitor cells, the expression of CXXC5 is changed dynamically with the stage of cell maturation, and knockdown of CXXC5 has been discovered to lead to a significant accumulation of immature cells [29]. Furthermore, CXXC5 plays an essential role in the development of monocytes; during myeloid differentiation, the down-regulation of CXXC5 was observed to result in a reduction in monocyte differentiation and an increase in granulocyte differentiation; knockdown of CXXC5 has been shown to enhance the expression of genes involved in cell cycle regulation, and an increased proportion of S-phase cells [86]. The loss, mutation, or functional defects in *CXXC5* are associated with a range of hematopoietic disorders, including abnormalities in proliferation and differentiation, and some studies have identified CXXC5 as an essential effector to promote terminal differentiation of cancer cells in the treatment of APL with ATRA [29].

Moreover, CXXC5 is also involved in the inhibition of cell proliferation and differentiation behaviors. CXXC5 is expressed in immature erythrocytes and inhibits cellular sensitivity to TGF-β signaling via Smad7, and CXXC5 knockdown has been observed to accelerate erythropoietin-driven maturation without affecting cell viability [52]. Furthermore, CXXC5 impedes mouse lung fibroblast (MLF) proliferation and transformation to myofibroblasts by inhibiting activation of the CD40/CD40L pathway and promotes apoptosis of MLFs, and CXXC5 overexpression has been discovered to inhibit the progression of bleomycin-induced lung fibrosis in mice [56].

### CXXC5 in apoptosis

CXXC5 regulates apoptosis through multiple pathways, including TNF-α, ATM/p53, FOXL2, and CD40/CD40L. CXXC5 is essential for the regulation of DNA damage-induced apoptosis, and in an in vitro experiment using wild-type MCF7 and HeLa cells, knockdown of CXXC5 influenced ATM phosphorylation and downstream p53 signaling [19]. Intriguingly, overexpression of CXXC5 in HEK293T cells did not result in enhanced p53 signaling, indicating that the regulation of ATM/p53 signaling by CXXC5 may be context-dependent or a necessary but not sufficient condition for it [53]. Similarly, CXXC5 expression was also found to correlate with TP53 mutation status in a population of BC patients, with high CXXC5 expression correlating with wild-type TP-53 and low CXXC5 expression correlating with TP53 mutation status, suggesting that high expression of CXXC5 may inhibit apoptosis in BC cells through other pathways [87].

In addition, CXXC5, as a protein partner of FXOL2, can promote apoptotic activity in wild-type FOXL2 KGN cells [60]. CXXC5 could induce apoptosis in TNF-α induced HEK293 cells by affecting the function of Smad3/4 proteins, and the regulation is dependent on the mitochondria-mediated apoptosis pathway; inhibition of the mitochondria-mediated apoptosis pathway by co-transfection of Bcl-2 was observed to inhibit CXXC5-induced apoptosis in primary rat cortical neurons [53]. Moreover, evidence indicates that CXXC5 can facilitate the TGF-β signaling pathway in hepatocellular carcinoma (HCC) by interacting with HDAC1, and its overexpression was discovered to result in apoptosis and cell cycle arrest in HCC [5]. In human malignant peripheral nerve sheath tumors (MPNSTs), CXXC5 is one of the downstream apoptosis mediators of KANK1 [40]. Furthermore, CXXC5 can induce apoptosis of MLFs by suppressing the CD40/CD40L pathway [56].

### CXXC5 in metabolism

CXXC5 can inhibit Cytochrome c oxidase Subunit 4 isoform 2 (COX4I2) promoter activity through an RBPJ-dependent pathway or independent pathway and interact with MNRR1 and RBPJ to affect COX4I2 expression and thus regulate cellular energy demand under different oxygen concentrations, which is a non-HIF1A-dependent ORE pathway [61]. In addition, CXXC5 has been shown to interact with CHCHD10, enhance its inhibition of oxygen-responsive element (ORE) transcriptional activity, and indirectly affect the composition and function of the mitochondrial respiratory chain [62]. CXXC5 is highly expressed inadipose tissues from obese type 2 diabetes (T2DM) patients. Blocking the CXXC5-Dvl interaction can improve the disease metabolic status of mice, reduce insulin resistance, inhibit adipocyte differentiation, facilitate the regeneration of pancreatic beta-cells, and enhance glucose homeostasis [71, 83]. Furthermore, a study has demonstrated that CXXC5 is associated with genes involved in insulin endocytosis, suggesting a potential role in the regulation of insulin resistance [88].

### CXXC5 in immunity and inflammation

CXXC5 appears to be repressed in non-immune tissues during inflammatory states. The expression of CXXC5 is downregulated in muscle tissues in COVID-19 induced cytokine storms and correlates with infection-induced muscle loss [45]. In the pathological state of periapical periodontitis due to *P. gingivalis* infection, CXXC5 expression is downregulated in c-cells and is associated with inhibition of differentiation of cementoblasts [33, 44, 46].

CXXC5 expression appears crucial for the immune response of immune cells. CXXC5 is highly expressed in mouse plasmacytoid dendritic cells (pDCs) and plays an important role in TLR7/9- and virus-induced IFN responses by recruiting Tet2 to maintain hypomethylation of specific CpG islands and stabilizing IRF7 expression. CXXC5-deficient mice are impaired in their early IFN responses and are susceptible to infection by herpes simplex virus and vesicular stomatitis virus [49]. In addition, the expression of CXXC5 is elevated in *E. granulosus*-infected mouse T follicular helper 2 (Tfh2) cells, which may be involved in the immune response they regulate [73]. CXXC5 interacts with SUV39H1, inducing trimethylation of H3K9. This process is involved in ThPOK-mediated inhibition of CD40L expression in CD8^+^ cytotoxic T cells [22]. Furthermore, the expression of CXXC5 was found to correlate with the infiltration of CD8^+^ T cells, resting memory CD4^+^ T cells, resting NK cells, activated dendritic cells, and memory B cells in chronic myeloid leukemia (CML), indicating that CXXC5 may be involved in anti-tumor immunity in CML [89].The identification of a heterozygous variant of CXXC5 in a patient with primary immunodeficiency disorders (PIDs) with decreased antibody production, progressive loss of B cells, and infections of the lungs and gastrointestinal tract also suggests an important role for CXXC5 in the immune system [90]. However, high expression of CXXC5 in immune cells is not always beneficial to the immune response of the organism. CXXC5 can enhance the activity of the *HIV-1* promoter, and its expression is upregulated in CD4^+^ T cells from patients with low-level viremia (LLV), which may be related to the activation of signaling pathways associated with viral replication and the low-level viremia state in these patients [55].

### CXXC5: from physiology to pathology

**Fig. 3:**
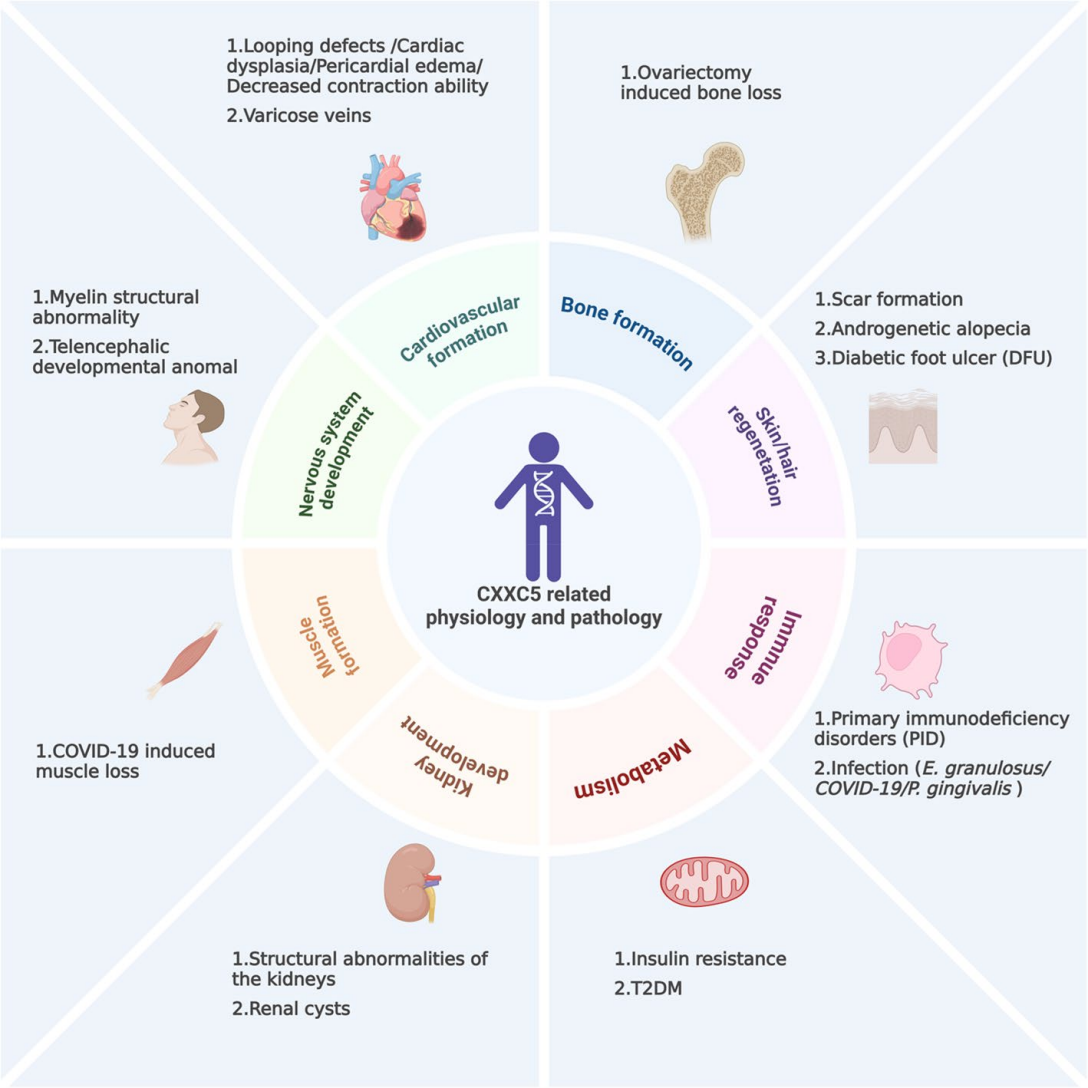
CXXC5 related phthological and physiological processes

As mentioned above, CXXC5 regulates various cellular behaviors, including proliferation, differentiation, and apoptosis, as well as physiological processes, such as growth and development, hair regeneration, and the immune response. Correspondingly, the abnormal expression or function of CXXC5 is closely associated with pathological conditions, including uncontrolled proliferation, restricted differentiation, immune dysregulation, and other abnormalities (shown in Fig. 3).

### CXXC5 in cancers

**Fig. 4:**
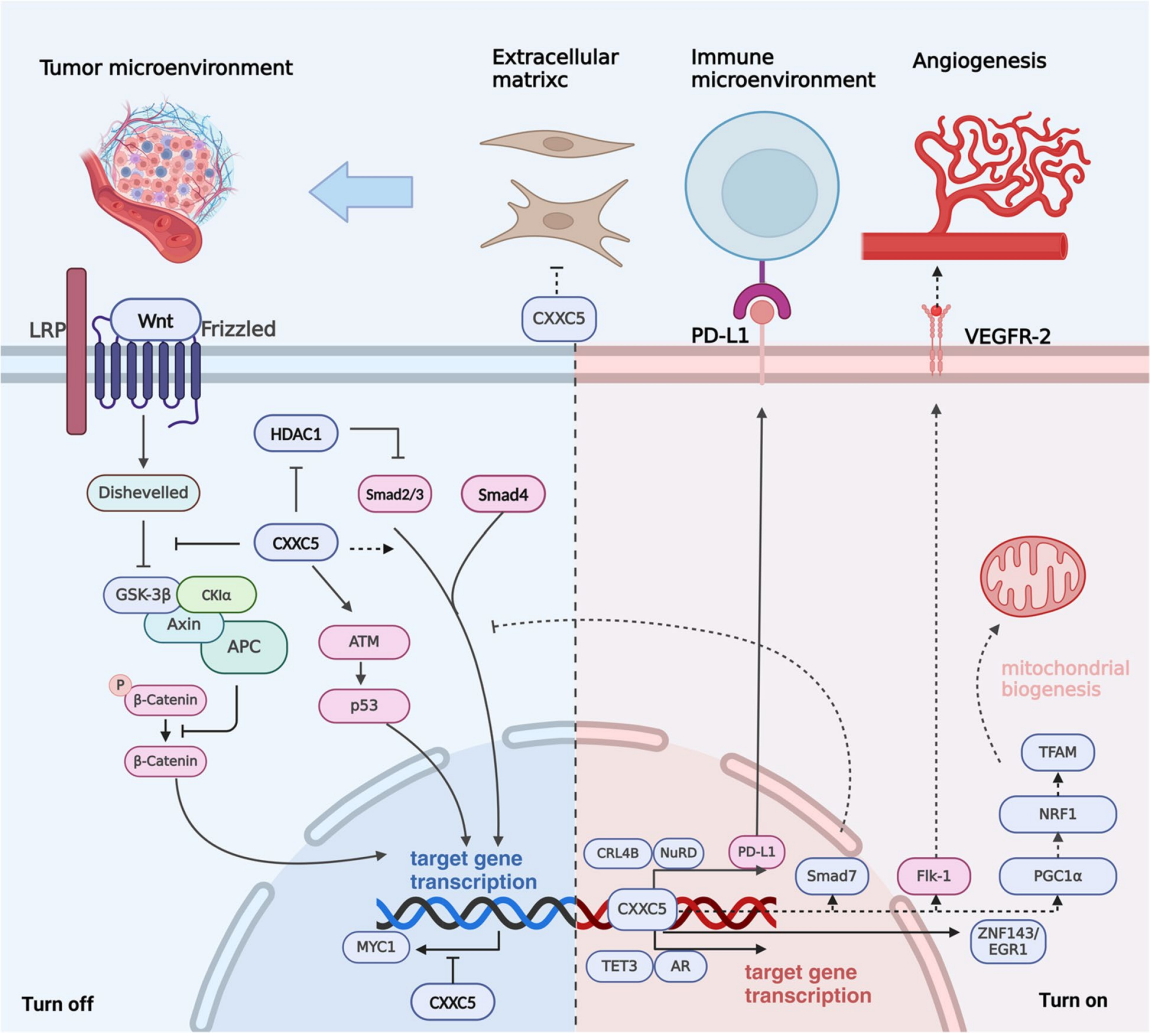
CXXC5 in cancer: turn on or off?

CXXC5 affects the activity of multiple signaling pathways, including TGF-β/BMP, Wnt/β-catenin, ATM/p53, and others [5, 19, 51]. Furthermore, CXXC5 influences the expression of its downstream target genes through epigenetic modification or transcriptional regulation. The complex signaling pathways and target genes associated with CXXC5 have different regulatory effects on tumors, which has resulted in the intricate and multifaceted role of CXXC5 in regulating tumorigenesis and cancer progression. CXXC5 is expressed at low levels in hematopoietic system tumors, gastric cancer (GC), and DIPG, indicating a potential tumor-suppressive role [6, 39, 47]. Conversely, CXXC5 is highly expressed in metastatic melanoma, thyroid carcinoma (THCA), BC, endometrial cancer (EC), PCa, and ovarian cancer (OC), suggesting a potential tumor-promoting function (shown in Fig. 4) [48, 87, 91, 92].

### Hematopoietic tumors

CXXC5 is intimately associated with the development of hematopoietic tumors. The expression of CXXC5 varies considerably between individuals in AML and acute lymphoblastic leukemia patient populations [93]. However, downregulated expression of CXXC5 is associated with low-risk abnormalities and higher overall survival in patients with newly diagnosed AML receiving intensive chemotherapy, indicating that CXXC5 may act as a tumor suppressor involved in tumor development [47, 89, 93].

Multiple factors play a role in the regulation of the expression or function of CXXC5 in hematopoietic tumors. The chromosomal location of the *CXXC5* gene (5q31.2) is a frequently deleted region in AML/MDS patients, which implicates that haploinsufficiency or functional defects in CXXC5 may be involved in the development of AML/MDS [12, 13]. In addition, in patients with AML, *MLL* rearrangements, t(8;21), and *GATA2* mutations have also been identified to downregulate the expression of CXXC5 [47]. In one study that included 22 AML and higher-risk MDS patients, all exhibiting a deletion of 5q, including CXXC5, 13 patients demonstrated CXXC5 expression levels below 50% of those observed in the standard control group. This finding suggests that factors other than haploinsufficiency may influence CXXC5 expression. Additionally, this study found that methylation and somatic mutations of *CXXC5* were rare in the AML/MDS patient population, with only 0/182 and 1/175 [94]. However, in a separate study that included 46 AML and 6 CD34^+^ samples, the promoter region of *CXXC5* was found to be highly methylated and associated with decreased expression in AML. Treatment with DAC inhibited methylation of the *CXXC5* promoter and promoted its expression in highly methylated cell lines U937 and NB4 [47]. Further data are required to elucidate whether methylation is involved in the regulation of CXXC5 in hematopoietic tumors. In myeloid malignancies, EZH2 regulates CXXC5 expression and influences disease development, and the mutation status of EZH2 affects CXXC5 expression [37].

CXXC5 exerts regulatory effects on the development of hematopoietic system tumors through several different mechanisms. One early study demonstrated that CXXC5, an essential effector in the treatment of APL with all-trans retinoic acid, could promote terminal differentiation of cancer cells [29]. In acute myeloid leukemia (AML), CXXC5 can inhibit leukemia cell proliferation and Wnt signaling and affect p53-dependent DNA damage response. Furthermore, the downregulation of CXXC5 was found to increase the susceptibility of AML cell lines to chemotherapy-induced apoptosis, and there were differences in the activity of apoptosis between primary human AML cells with high expression of CXXC5 and low expression, suggesting that CXXC5 may influence tumor cell development by regulating cellular drug resistance and apoptosis [47, 93]. The expression of CXXC5 in CML was related to the P53 pathway, DNA repair, MYC targets, and apoptosis, which may be involved in the regulation of CML cell proliferation. Furthermore, the expression of CXXC5 was associated with immune cell infiltration, suggesting a potential involvement of CXXC5 in anti-tumor immunity in CML [89].

### HCC

The function of CXXC5 in HCC development remains controversial. One study revealed that CXXC5 expression was reduced in most HCC tissue samples compared with normal tissues, and the competitive binding of CXXC5 to HDAC1 was found to upregulate TGF-β signaling and thus induce cell cycle arrest and apoptosis in HCC cells under in vitro test conditions [5]. However, it has also been found that over-regulation of CXXC5 expression in Hep3B cells promotes the growth, migration, and invasion of HCC cells [35]. Hepatic fibrosis represents a critical step in the transformation of normal liver cells to HCC [95], and CXXC5 expression is down-regulated during the activation of hepatic stellate cells, which inhibits the expression of the proto-oncogene MYCL1 by binding to the promoter region of the *MYCL1* gene. Deletion of CXXC5 leads to hepatic stellate cells activation and promotion of hepatic fiber progression [9].

### BC

CXXC5 plays a role as a tumor-promoting factor in BC. The expression of CXXC5 is significantly upregulated in patients with advanced BC, and its high expression correlates with higher tumor grade and poorer prognosis in BC patients [31, 87]. E2 plays a vital role in the homeodynamic regulation of breast tissue, and the ERα is the primary transcript expressed in breast tissues, and ER-ERα signaling can directly promote the expression of CXXC5 [16, 24]. Abnormalities in E2 are strongly associated with the overexpression of CXXC5 and the development of BC. In the BC patient population, CXXC5 is highly expressed in patients with ER+ BC compared to basal-like and triple-negative BC, and it has a poor prognosis [24, 96].

The mechanism by which CXXC5 promotes BC development has not been fully clarified. One study found that CXXC5 inhibits the TSC1/mTOR signaling pathway by interacting with CRL4B and NuRD, which promotes BC development and PD-L1-mediated immune escape [31]. Another study found that high expression of CXXC5 was associated with wild-type TP53 in BC, suggesting that high expression of CXXC5 may inhibit apoptosis and promote tumor development through other mechanisms [87].

### GC

CXXC5 is a potential tumor suppressor in GC, and CXXC5 expression is downregulated in GC tissues and cells [39, 97]. Mechanistically, KANK1 plays a tumor suppressor role in various tumors and positively regulates the expression of CXXC5 [98]. The downregulation of KANK1 expression in GC is one of the factors leading to the downregulation of CXXC5 in GC. In addition, CXXC5 promotes apoptosis and inhibits EMT, migration, and invasion of GC cells by inhibiting Wnt/β-catenin/Axin2 in GC tissues [39].

### PCa

CXXC5 is highly expressed in PCa compared to normal prostate tissue, and high expression of CXXC5 is associated with poorer overall survival in PCa patients treated with AR signaling inhibitors, including ENZ and ABI [27, 92, 99]. CXXC5 binds directly to unmethylated CpG and subsequently recruits TET2 and AR to non-canonical AR target gene loci, promoting transcription of downstream target genes as well as ENZ resistance and growth of CPRC cells [27]. However, it has also been found that *CXXC5* is frequently hypermethylated in PCa compared to normal tissues, and PCa patients with low CXXC5 expression have a higher risk of recurrence compared with those with high expression [43, 100]. Further investigation is required to elucidate the mechanism of CXXC5 in PCa development.

### Esophageal squamous cell carcinoma (ESCC)

In esophageal squamous cell carcinoma (ESCC), upregulated miR-32 can target the 3'-UTR of CXXC5 to inhibit CXXC5 expression and is associated with increased capacity of migration, invasion and adhesion in ESCC [38].

### THCA

CXXC5 expression is upregulated in patients with malignant THCA [87]. Another study found that *CXXC5* showed a hypomethylated state in THCA [42]. The specific mechanisms of hypermethylation status of *CXXC5* in THCA need to be further investigated.

### DIPG

CXXC5 expression is reduced in DIPG, and high CXXC5 expression is associated with a better prognosis in DIPG patients. In the context of DIPG cells, its deletion or down-regulation promotes DIPG cell proliferation and reduces apoptosis [6].

### OC

CXXC5 is an important tumor-promoting factor in OC. A study reported that CXXC5 was markedly expressed in OC and was associated with a poor prognosis. Regarding mechanisms, CXXC5 is induced by HIF1A in hypoxic environments and promotes OC proliferative activity by facilitating the expression of its downstream transcription factors ZNF143 and EGR1 [48].

### EC

In EC patients, high expression of CXXC5 was associated with poor overall and disease-free survival, as well as an elevated risk of recurrence. Inhibition of cell metabolism and colony formation, as well as elevated caspase3/7 activity, could be observed in uterine plasmacytoid carcinoma cell lines knocked down for CXXC5, suggesting that CXXC5 may promote tumor progression by inhibiting apoptosis of plasmacytoid endometrial carcinoma cells and promoting their proliferation and invasion to facilitate tumor development [91].

### CXXC5 in other diseases

The involvement of CXXC5 in the development of additional diseases has been reported. CXXC5-MH1 fusion was found in a patient with astroblastoma [14, 15]. Moreover, the expression of CXXC5 is downregulated in patients with diminished ovarian reserve (DOR), and the exact role of CXXC5 in DOR remains to be elucidated [101]. Additionally, CXXC5 exerts an antifibrotic effect by inhibiting CD40/CD40L during the development of bleomycin-induced pulmonary fibrosis in mice [56]. Furthermore, CXXC5 is involved in the pathogenesis of Alzheimer's disease (AD). CXXC5 is overexpressed in the tissues of AD patients and 5xFAD transgenic mice, accompanied by the repression of the Wnt/β-catenin signaling pathway and its target genes related to AD. Inhibition of the CXXC5-Dvl interaction resulted in a notable improvement in the Alzheimer's disease status of the 5xFAD mice [77].

## Potential diagnostic and therapeutic value of CXXC5

In terms of tumor therapy, it is currently predictable that CXXC5 has essential value in tumor diagnosis. In the context of BC diagnosis, CXXC5 expression is significantly elevated in patients with advanced stages of BC, and its high expression correlates with higher tumor grade and poorer prognosis in patients. Furthermore, CXXC5 is indicative of different tumor types and is highly expressed in ER+ BC patients compared to basal-like and triple-negative BC [96]. It is noteworthy that CXXC5 is not differentially expressed in the BC population; instead, its transcription regulations demonstrate quantitative associations with BC in diversified cohorts [102]. CXXC5 has a high potential for application in the diagnosis of tumors, but there are also difficulties; as it was mentioned above, CXXC5 might play different roles at different stages of HCC progression, and its expression is altered accordingly, which may make the process from theory to realizing the value of CXXC5 in tumor diagnosis difficult.

CXXC5 also has high application value in tumor therapy. It may be a promising approach to target the oncogenic or oncostatic effects of CXXC5 in tumors through gene silencing or activation by RNA interference, gene editing, and other methods. However, like similar attempts with other molecules, this strategy still faces various problems from theory to clinical practice, including delivery strategy, accuracy, and safety. Furthermore, the intricate mechanism of CXXC5 in cancer regulation offers numerous potential targets for small molecule peptides, and it is a highly feasible strategy to achieve therapeutic goals by designing small molecule peptides to target the stability, cellular localization, and site of CXXC5 action. Regardless of the strategy, it is necessary to consider the dual role of CXXC5 in cancer progression when targeting it for therapeutic purposes. The impact of CXXC5 in cancer results from its dual mechanism of action (oncogene and tumor suppressor), which may reduce its inhibitory potential when attempting to inhibit its cancer-promoting activity. Consequently, combination therapy targeting multiple sites would hopefully be a more efficacious approach for certain cancer therapy.

In the remaining diseases, the current therapeutic strategies targeting CXXC5 primarily focus on elucidating the interaction between CXXC5 and Dvl. The therapeutic effects of blocking CXXC5-Dvl interactions have been demonstrated in animal models for treating AD, skin healing, angiogenesis, bone formation, and hair regeneration [25, 26, 46, 57–59, 63, 71, 72, 76–80, 82, 83, 103–105]. Targeting the CXXC5-Dvl interaction has unique therapeutic advantages and may have broader clinical applications. The current prescription of parathyroid hormone (PTH)-based-anabolic drugs for the treatment of osteoporosis, for instance, is limited by factors such as high cost and risk of osteosarcoma. Furthermore, the use of anabolic antibody-based medicines may be constrained by macromolecules. The blocking of the interaction between CXXC5 and Dvl has been demonstrated to significantly promote osteoblast differentiation and inhibit bone loss, which has potential advantages in future applications [72, 106, 107] (summarized in Table 4).

**Table 4:**
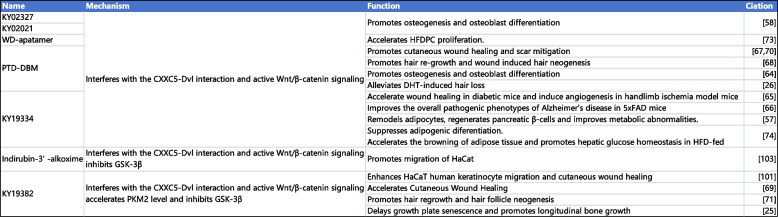
Drugs and chemicals targeting CXXC5 [25, 26, 57–59, 63, 71, 72, 76–80, 82, 83, 103]

*Abbreviations*: *PTD-DBM *Protein transduction domain-fused Dvl-binding motif

## Perspectives

In summary, CXXC5 is a member of the ZF-CXXC protein family, acting as a signal integrator and information transfer in the cellular signaling network. Multiple factors affect the expression or function of CXXC5, resulting in the alterations in cell proliferation, differentiation, apoptosis, metabolism, and other cellular behaviors through transcriptional regulation, epigenetic modification, and the modulation of cellular signaling pathways. CXXC5 regulates physiological processes such as angiogenesis, hair regeneration, and growth and development, and its aberrant expression or function is linked to various diseases, including cancer. Consequently, CXXC5 has potentially clinical value as a future diagnostic indicator or therapeutic target.

Nevertheless, further investigation is required to elucidate the relationship between the structure and function of CXXC5. The ZF-CXXC domain is central to the mechanism of CXXC5; however, this structural domain is conserved within the ZF-CXXC family, and how the specificity of CXXC5 action is achieved deserves further investigation. Furthermore, the relationship between ZF-CXXC and other domains requires elucidation. As previously stated, some of the functions have been found to be associated with the NLS domain, the DBD, and the intermediate region (residues 101–200) of CXXC5 [19–21]. However, it remains unclear whether the ZF-CXXC domain can exert a canonical regulatory effect independently and whether these domains can exert a non-canonical effect independently or independently of the ZF-CXXC domain.

As previously stated, the function and expression of CXXC5 are regulated by multiple upstream factors. It seems reasonable to assume that similar factors may influence CXXC5 expression, including progestin (given that CXXC5 is an essential effector of E2 and the close association of progestin with E2 function) [16, 24, 108], follicle-stimulating hormone/luteinizing hormone (given the down-regulation of CXXC5 in the DOR and the close association of follicle-stimulating hormone and luteinizing hormone with ovarian development and the pathological state of the DOR) [101, 109], as well as other signaling pathways, and others. Furthermore, the regulatory function of CXXC5 in more physiological and pathological processes can be inferred from the available evidence. For example, as an important effector of E2, CXXC5 is involved in E2-mediated growth plate senescence and BC development. CXXC5 may also be essential in other E2-ERα-mediated physiological and pathological processes [16, 24, 25]. Additionally, the relationship between CXXC5 and E2 and other estrogen receptors merits further investigation.

Context-dependence is an important feature of CXXC5 as a signal integrator and information transfer in cellular signaling networks, as reflected in both the regulation of CXXC5 expression by upstream signals and the regulatory function of CXXC5 itself. In the HL-7702, Hep3B, HepG2, and Huh7 cell lines, TGF-β-exposure results in enhanced CXXC5 expression; however, no such effect is observed in the MHCC97L or MHCC97H cells, reflecting the context-dependent regulation of CXXC5 expression by upstream factors [5]. Similarly, the previously mentioned regulation of Smad7 by CXXC5 can be also considered context-dependent regulation [52, 69]. Furthermore, CXXC5 significantly enhances the transcriptional activity of the *HIV-1* promoter but exerts a repressive influence on the transcriptional activity of the *SV40* and *CMV-IE* promoters [55]. A more nuanced comprehension of this context-dependence can facilitate a more profound grasp of CXXC5 function in disparate cellular milieus and offer a foundation for subsequent interventions targeting CXXC5.

Interestingly, CXXC5 sometimes exhibits "opposite" regulatory roles in the physiological and pathological processes. CXXC5 promotes angiogenesis by promoting Flk-1 expression, but the interaction of CXXC5 with Dvl inhibits angiogenesis in the DFU mouse model [4, 76]. More similar "opposite" regulatory effects can be observed in cancers. CXXC5 has been found to act as an oncogene in some cancers and as a tumor suppressor in others. Furthermore, CXXC5 even displays "opposite" regulatory functions at different stages of the same cancer type's development. For instance, CXXC5 can inhibit the process of liver fibrosis but promote the development of HCC (another study has suggested that CXXC5 can inhibit the growth of HCC) [5, 9, 35, 95]. In the context of therapies targeting CXXC5, this "opposite" regulatory effect needs to be focused on. In angiogenesis-related diseases, targeting CXXC5 expression or CXXC5-Dvl interaction alone may prove ineffective due to the negative feedback regulation of the Wnt/β-catenin signaling pathway by CXXC5. In addition, CXXC5 itself has cancer-inhibitory potential. Activation of this inhibitory potential in some cancers where CXXC5 acts as an oncogene with high expression may restrain cancer development more efficiently.

Currently, the regulatory role of CXXC5 in cancer development focuses on the effects of CXXC5 on cancer cell proliferation, invasion, resistance to apoptosis, and others. However, from the point of view regarding CXXC5-regulated physiological processes, CXXC5 may have a more multidimensional impact on cancer development. Since CXXC5 regulates immune response, angiogenesis, and fibroblast differentiation, it is not difficult to speculate that CXXC5 may play an essential role in the tumor microenvironment that promotes or inhibits carcinogenesis. In addition, the association of CXXC5 with mitochondrion generation and adipocyte cell metabolism, as well as the critical role of CXXC5 in epigenetic modification, as mentioned earlier, suggests that CXXC5 may be involved in metabolic reprogramming and non-mutational epigenetic reprogramming processes in tumor development. The additional regulatory roles of CXXC5 on cancer development deserve further investigation.

There are also several areas of concern. CXXC5 is important for ATM phosphorylation and ATM/p53 signaling, but overexpression of CXXC5 cannot enhance p53 signaling, indicating the regulation of ATM/p53 signaling by CXXC5 may be context-dependent or a necessary but not sufficient condition for it [19, 53]. Furthermore, a study has demonstrated a correlation between the methylation level of the *CXXC5* and paternal longevity. The CXXC5 gene exhibits lower methylation levels in offspring with more extended paternal longevity, which may provide new insights into the heritability of longevity and cross-band epigenetic regulation [110]. Moreover, CXXC5 expression can be reduced by vitamin D treatment, and it has been found that CXXC5 can act as a vitamin D receptor (VDR) interacting protein and stimulate the vitamin D receptor-mediated transcription of specific VDREs, indicating that CXXC5 may have a feedback role in vitamin D signaling [31, 64].

## Data Availability

No datasets were generated or analysed during the current study.
